# Effects of vitamin D supplementation on maximal strength and power in athletes: a systematic review and meta-analysis of randomized controlled trials

**DOI:** 10.3389/fnut.2023.1163313

**Published:** 2023-09-29

**Authors:** Marco Sist, Lu Zou, Stuart D. R. Galloway, Nidia Rodriguez-Sanchez

**Affiliations:** ^1^Faculty of Health Science and Sport, Physiology, Exercise and Nutrition Research Group, University of Stirling, Stirling, United Kingdom; ^2^AstraZeneca, London, United Kingdom

**Keywords:** cholecalciferol, ergocalciferol, muscle function, 1RM test, vertical jump

## Abstract

**Background:**

Vitamin D is thought to be a powerful modulator of skeletal muscle physiology. However, available data on the effects of vitamin D supplementation on muscle function in athletes are limited and with mixed results. This meta-analysis therefore, aimed to quantitatively summarize the up-to-date literature assessing the effects of vitamin D supplementation on muscle strength and power in athletes.

**Methods:**

Sport Discus, PubMed, Cochrane Library and Web of Science were searched to identify randomized controlled trials (RCTs) that used one-repetition maximum (1RM) tests to assess maximal strength, and vertical jump to assess muscle power in athletes. The Cochrane Risk of Bias tool was used to evaluate the included RCTs for sources of bias. The standardized mean difference (SMD) was used as the effect size, interpreted together with its 95% confidence intervals (CI). The effect sizes were calculated on the changes from baseline between vitamin D and placebo groups for maximal strength results by upper body and lower body, and for power results.

**Results:**

Eleven RCTs involving 436 athletes were included. The results indicated that if baseline serum 25(OH)D concentration was < 75 nmol/L, the treatment had a small effect on upper body muscle strength [SMD 0.25, 95% CI: (−0.44, 0.95), *p* = 0.47] and on lower body muscle strength [SMD 0.26, 95% CI: (−0.13, 0.65), *p* = 0.19]; if the baseline serum 25(OH)D concentration was ≥ 75 nmol/L, the treatment had a trivial effect on muscle power [SMD 0.15, 95% CI: (−0.42, 0.72), *p* = 0.61].

**Discussion:**

This meta-analysis demonstrated that there is not a statistically significant effect of vitamin D supplementation on improving maximum strength and power, but highlights that further research is required addressing the key limitations in previous studies before definitive conclusions can be made.

## Introduction

Vitamin D is a fat-soluble pro-hormone that occurs in two forms, cholecalciferol (vitamin D_3_) and ergocalciferol (vitamin D_2_). Cholecalciferol can be obtained from sunlight exposure of the skin, with a small amount coming from the diet (fatty fish, eggs, and liver), while ergocalciferol can be obtained by UVB irradiation of the ergosterol in plants and fungi (e.g., mushrooms). Both vitamin D_2_ and D_3_ undergo hydroxylation in the liver, where they are converted into 25-hydroxyvitamin D [25(OH)D, Calcidiol]. 25(OH)D is further hydroxylated in the kidney to form 1,25-hydroxyvitamin D (calcitriol), a biologically active metabolite, which then binds to vitamin D receptors (VDR) at target tissues (bone, immune system cells, muscle cells) and exerts its function (1). Vitamin D plays a central role in calcium and phosphate homeostasis and is essential for development and maintenance of healthy bone. It enhances intestinal calcium and phosphate absorption, stimulates osteoclast differentiation and calcium reabsorption form bone, and promotes mineralization of the bone matrix (2). In the immune system cells, 1,25-hydroxyvitamin D enhances the innate immune response primarily via its ability to stimulate cathelicidin, an antimicrobial peptide important in defense against invading organism, whereas it inhibits the adaptive immune response primarily by inhibiting the maturation of dendritic cells (DC) important for antigenic presentation, reducing T cell proliferation, and shifting the balance of T cell differentiation from the Th1 and Th17 pathways to Th2 and Treg pathways (2). At muscle cell level, vitamin D is thought to be a modulator of skeletal muscle physiology through both genomic and non-genomic events (3, 4). The genomic events occur through modulation of gene transcription and protein synthesis, influencing muscle cell proliferation and differentiation, particularly in fast-twitch fibers (5). The non-genomic events responses involve calcium and phosphate transport by muscles through cell membranes, directly impacting muscle contraction (6). Moreover, there is now evidence that 25(OH)D accumulates in skeletal muscle cells, which may provide a functional store during winter and regulate its concentration in blood when vitamin D supply is low (7).

Despite its well-recognized importance on muscle function, large portions of athletic populations are vitamin D deficient, with the risk significantly increasing in higher latitudes, in winter and early spring season, and for indoor sport activities (8). However, what exactly constitutes vitamin D deficiency is subject to intense debate. Even if current evidence suggests that a serum 25(OH)D concentration below 75 nmol/L might be considered deficient, at least in white male athletes (9), it is not clear if low total 25(OH)D levels might uniformly indicate vitamin D deficiency in athletes with different ethnicity. In fact, levels of total 25(OH)D and vitamin D-binding protein (VDBP) are lower in blacks than in whites, while levels of bioavailable 25-hydrosxyvitamin D are equivalent, indicating 1,25(OH)D as a possible better marker of the vitamin D status (10). Some data shows that vitamin D deficiency negatively affects muscle function, contributing to proximal muscle weakness with a reduction in type II muscle fibers diameter (11). Since greater muscle function is strongly associated with improved force-time characteristics (12), and with a reduced incidence of injuries (13, 14), research on the effects of vitamin D on muscle strength in athletic populations has been gathering interest. However, even if vitamin D supplementation is considered effective in improving vitamin D status (15), available data on the effects of vitamin D supplementation on maximal strength and power in athletes are limited and with mixed results (16, 17). In a recent meta-analysis summarizing the evidence of vitamin D effect on muscle strength and power in athletes, the researchers highlighted that vitamin D supplementation had a significant effect on increasing lower body muscle strength, but not on increasing upper body muscle strength or muscle power (16). The reason for this difference remains unclear. Another meta-analysis (17) investigating the effect of vitamin D_3_ on serum 25(OH)D concentration and strength in athletes showed that the overall effect of vitamin D_3_ administration on muscle strength was not statistically significant. These reviews however, present some limitations. First, the effect sizes were calculated by comparing the results at follow-up point, and not on the changes in means and SD from baseline between vitamin D and placebo groups; second, the sample size used in the trials included in these meta-analyses is small [219 subjects in Zhang et al. (16), and 163 subjects in Han et al. (17)]; third, there is high heterogeneity between-study baseline serum 25(OH)D concentration, with some of the included studies where participants have a serum 25(OH)D concentration below 75 nmol/L and others greater than 75 nmol/L; finally, the tests used to assess muscle strength are inconsistent across the included studies, varying from isokinetic and isometric tests to 1 repetition maximum (1RM) tests. Although dynamometry is considered the “gold standard” for assessing muscle strength *in vivo*, 1RM tests reflect the kind of dynamic ability necessary in sport, and they are the choice for most strength and conditioning professionals for athletic populations (18).

Considering the conflicting results and limitations of these meta-analyses, this study aimed to quantitatively summarize the up-to-date literature assessing the effect of vitamin D supplementation on muscle strength and power in athletes, selecting randomized controlled trials (RCTs) that used standard measurements for maximal strength (1 RM test for multi-joint exercises) and power (any vertical jump).

## Methods

This systematic review and meta-analysis was conducted following PRISMA 2020 (Preferred Reporting Items for Systematic Reviews and Meta-Analysis) statement (19) to ensure rigorous methodology and reporting. Marco Sist, Lu Zou, Stuart D. R. Galloway and Nida Rodriguez-Sanchez were the investigators completing the review.

### Eligibility criteria

The PICO approach was used to frame the research question as follow: Population (P) was defined as male and female athletes, younger than 35 years, with no restrictions on sport or competitive level; Intervention (I) was oral administration of vitamin D_2_ or D_3_, not limited to any dosage or duration; Comparison (C) was between intervention and placebo; Outcomes (O) were maximal strength and power (20). Only published randomized controlled trials (RCTs) conducted among athletes were included. Non-randomized trials and non-athletes-related trials were excluded. Research was also excluded if including athletes with chronic illness, injury or impairment, if muscle strength was not assessed by 1 RM tests, or muscle power by jump tests, and if intervention included multivitamin supplementation.

### Information sources and search strategy

A literature search of Sport Discus, PubMed, Cochrane Library, and Web of Science from inception to 13th March 2022 was accomplished. Google Scholar was also searched for the gray literature. The following search terms and medical subject headings (MeSH) were used: vitamin D, ergocalciferol, cholecalciferol, maximal strength, muscle power, bench press, squat, deadlift, jump, swimming, soccer, rugby, basketball, rowing, running, football, skiing, tennis, cyclist, team sports, military personnel, military training, tactical training. The complete search strategy is attached in Supplementary material 1. The search was updated on 10th May2023 to retrieve the most recent publications.

### Selection process

The search results were exported to Covidence (21), the Cochrane systematic reviews production tool for quality assessment and data extraction, and merged; duplicates were removed automatically. Titles and abstracts were reviewed by MS, and eligible trials were selected for full texts examination. NR-S and SG supervised the whole process.

### Data collection process

Data were sought independently by MS for 1 RM tests on any upper or lower body multi-joint exercise and for lower body muscle power assessed by any vertical jump, and were collected using tables presented in the studies, extrapolated from figures or requested directly to the authors by email, when tables or data description were missing. NR-S and SG supervised the whole process.

### Data items

The following characteristics of the included RCTs were collected: studies’ location, latitude and season, sport activity, training environment, participants’ age, race or ethnicity, sample size at baseline and follow-up for intervention and control groups, participants lost to follow-up, and % of males. The treatment information of vitamin D_2_ and D_3_ including serum concentration at baseline and follow-up, dose, study duration, type, product, methods of analysis were extracted and tabulated. The outcomes for 1 RM test or power test were reported as mean ± SD and N at baseline and follow-up endpoints, for both intervention and control groups. The reported SE was converted to SD by multiplying SE and the square root of the sample sizes. Data reported as median and percentiles were converted into mean ± SD (22). The weighted mean was calculated if data were reported separately for males and females (23). The timing and dosage of vitamin D supplementation varied between trials, and were converted into a weekly dosage with international units (IU). All serum 25(OH)D concentrations reported in ng/mL were converted into nmol/L for consistency, where 1 ng/mL equals to 2.5 nmol/L. In addition, all jump test values reported in inches were converted into centimeters, where 1-inch equals 2.54 centimeters. During data extraction, it was noted that durations of interventions among different studies were 4, 6, 8, 12, and 16 weeks. Therefore, trials were stratified by endpoint ≤ 16 weeks. It was also noted that baseline serum 25(OH)D concentration was ≥ 75 nmol/L in three studies, and < 75 nmol/L in eight studies; therefore, trials were stratified into two subgroups by baseline vitamin D sufficiency (≥ 75 nmol/L and < 75 nmol/L). One study (24) presented multiple intervention groups (20.000 and 40.000 IU of vitamin D per week vs. placebo). In order to have two independent comparisons and overcoming a unit-of-analysis error, the placebo group was split into two groups with a smaller sample size (25). Several studies presented multiple outcomes (1 RM tests for both upper and lower body muscle strength or multiple 1 RM tests for upper or lower body muscle strength or muscle power). In order to separate the upper and lower body, analyses have been applied independently. Whenever a study presented multiple outcomes for upper or lower body muscle strength or muscle power, a single outcome was selected based on the 1RM test or power test most commonly used in the other studies (26).

### Data synthesis

All analyses were conducted using Review Manager software 5.4 (27). Considering that different exercises were employed to assess maximal strength and power, the standardized mean difference (SMD) was used as the effect size, interpreted together with its 95% confidence intervals (CI). The effect sizes were calculated on the changes from baseline between vitamin D and placebo groups for serum 25(OH)D concentration results, for maximal strength results by upper body and lower body and for power results, and were considered as: “trivial” (<0.2), “small” (≥0.2, <0.5), “moderate” (≥0.5, <0.80), large (≥0.8) (28). Pooled estimates were obtained by a random effect model with inverse variance weighting. Between-study heterogeneity was assessed by calculating *I*^2^ statistics (*I*^2^ < 40% as low, 30–60% as moderate, 50–90% as substantial and 75–100% as considerable heterogeneity) (29). Sensitivity analysis was conducted when there was evidence of heterogeneity by removing studies with higher\lower weights to evaluate the robustness of the results.

### Study risk of bias assessment

The Cochrane Risk of Bias tool (30) was used to evaluate the included RCTs for sources of bias in the following domains: random sequence generation, allocation concealment, blinding of participants and personnel, blinding of outcome assessment, incomplete outcome data, selective reporting, and other bias (Figure 2). Reporting bias have been assessed by controlling for differences between what was reported in “results” session with was claimed in “methods” session because of the lack of access to protocols. The trials were graded as “low risk” if adequately described, “high risk” if not described, or “unclear risk” if inadequately described. A funnel plot was used for visual assessment of publication bias.

### Certainty assessment

We used the Grading of Recommendations, Assessment, Development and Evaluation (GRADE) system to assess evidence quality (31), which includes five domains, risk of bias, inconsistency, indirectness, imprecision, and publication bias. The quality of evidence for each outcome was graded as “high,” “moderate,” “low,” or “very low.” MS assessed the reporting, methodological and quality of evidence; SG and NR-S supervised the whole process.

## Results

### Study selection

**FIGURE 1:**
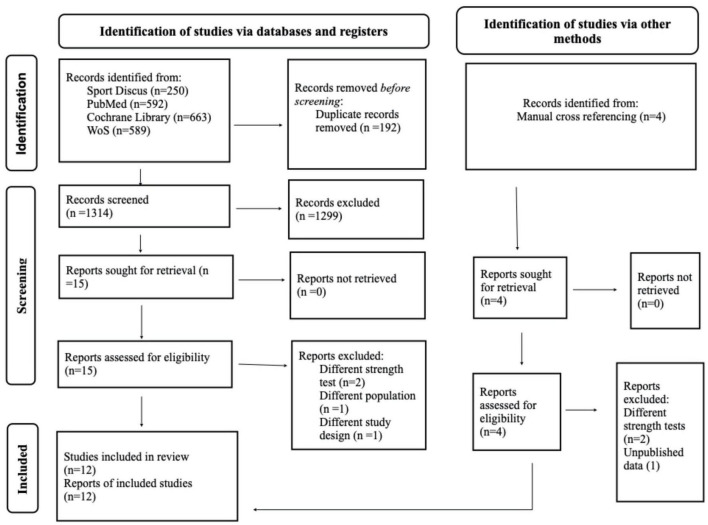
PRISMA 2020 flow diagram for the literature search and selection process.

After reviewing 1,279 titles and abstracts, 19 articles were selected for full-text review. Of the 19 articles, twelve RCTs were included in this review (24, 32–42), and seven studies were excluded because they didn’t use 1RM tests to assess maximal strength (43–46), included master athletes (47), used different study design (48), or were not published (49) (Figure 1).

### Study characteristics

The location of the studies varied from the UK (four studies), to the USA (three studies), to New Zealand, Tunisia, Poland, Republic of Korea, and Iran (one study each). All studies except one (32) reported the period of the year that the intervention trial was performed: eight studies were conducted during winter, two during autumn, one in autumn\winter and one in winter\spring. A total of 480 athletes were engaged in different sports: football, Gaelic football, futsal, soccer, rugby, taekwondo, swimming, gymnastic, dancing, and NASCAR pit crew. Seven out of twelve studies included males only and five studies included a mixed population. Race or ethnicity of participants was reported in only three studies: 18 white and 1 black athletes in one study (39), European, New Zealand Maori and Pacific athletes in another study (35), and African American, Caucasian, and Hispanic athletes in the third study (50). Mean age varied from 11 ± 2 years old in young soccer players (33) to 24 ± 5 in professional football and futsal player (32) (Table 1).

**TABLE 1:** Characteristics of the included RCTs.

References	Location, latitude and season	Sport activity	Training environment	Age (years)	Randomized (VIT D/PLA)	Analyzed (VIT D/PLA)	Lost to follow-up	% males	Ethnicity\ Race
(32)	Iran, 35.6°N, NR	Football, futsal	Outdoor, indoor	24 ± 5	35/35	35/34	1 (1.4%)	52.2%	N\S
(33)	Tunisia, 36°N, Winter	Football	Outdoor	11 ± 2	20/20	19/17	4 (10%)	100%	N\S
(24)	UK, 53°N, Winter	Football, Rugby	Outdoor	21 ± 1	10/10/10	9/10/6	5 (16.7%)	100%	N\S
(34)	UK, 53°N, Winter	Soccer	Outdoor	18 ± 5	5/5	5/5	0 (0%)	100%	N\S
(35)	New Zealand, 45°–46.5°S, Autumn	Rugby	Outdoor, indoor	21 ± 3	29/29	28/29	1 (3.4%)	100%	E, NZM, P
(36)	Poland, 54.3 N°, Winter	Soccer	NR	17 ± 1	20/16	20/16	0 (0%)	100%	N\S
(42)	South Korea, 33°29’ N, Winter	Taekwondo	Indoor	20 ± 1	22/11	20/15	9 (20.5%)	60%	N\S
(37)	USA, 35°N, Winter	NASCAR pit crew	Indoor	27 ± 1	15/15	13/15	2 (6.7%)	100%	N\S
(39)	USA, 37°N, Autumn	Swimming	Indoor	20 ± 1	10/9	10/9	0 (0%)	31.6%	18 W, 1 B
(38)	USA, 35°N, Winter	Mixed Sports	Outdoor, indoor	16 ± 1	17/17	17/17	0 (0%)	100%	AA, C, HA
(40)	UK, 55°N, Autumn/Winter	Gaelic Football	Outdoor	20 ± 2	22/20	22/20	0 (0%)	42.9%	N\S
(41)	UK, 51.5°N, Winter/Spring	Dancing	Indoor	17 ± 1	45/22	45/22	0 (0%)	56.7%	N\S

Data are mean ± standard deviation unless stated otherwise. N\S, not specified, E, European; NZM, New Zealand Maori; P, Pacific; AA, African American; C, Caucasian; HA, Hispanic; W, White; B, Black.

Ten studies administered vitamin D_3_ as an intervention, and two studies used vitamin D_2_ (37, 50). Supplement dosages ranged from a single dose of 120.000 IU (41) to 50.000 IU per week for 8 weeks (32). The duration of the studies varied from 6 (37, 50) to 16 weeks (41).

Maximal strength was measured by 1 RM tests for Bench Press, Chin-Up, Bench Pull, Leg Press, Squat, and Deadlift in five studies; muscle power was measured by Vertical Jump, Squat Jump, CMJ, Single leg Hop, and Standing Broad Jump in ten studies (Table 2).

**TABLE 2:** Baseline measurements of the included RCTs.

References	Baseline 25(OH)D (nmol/L) - Vit D Group	Baseline 25(OH)D (nmol/L) - PLA Group	Dose (IU/Week)	Study duration	Type	Product	25OH(D) method of analysis	Outcome (unit)
(32)	68.8 ± 44.8	61.0 ± 31.8	50.000	8 weeks	D3 solution	Zahravi Pharmaceutical Company	ECLIA analyzer (Liaison, DiaSorin, Italy)	Leg Press (kg), Vertical Jump (cm)
(33)	31.0 ± 9.7	30.3 ± 11.3	16.700	12 weeks	D3 solution	Bouchara Ricordatti	CLIA analyzer (Liaison, DiaSorin, USA)	Vertical jump (cm), standing broad jump (cm), Triple-hop (cm)
(24)	53.0 ± 26.0	52.0 ± 27.0	20.000	12 weeks	D3 capsules	Biotech Pharmacal Inc., Arizona, USA	HPLC-MRM (Becton Dickinson, Oxford, UK)	Bench Press (kg), Leg Press (kg), Vertical Jump (cm)
(34)	51.0 ± 26.0	52.0 ± 27.0	40.000	12 weeks	D3 capsules	Biotech Pharmacal Inc., Arizona, USA	HPLC-MRM (Becton Dickinson, Oxford, UK)	Bench Press (kg), Leg Press (kg), Vertical Jump (cm)
(34)	29.0 ± 25.0	53.0 ± 29.0	35.000	8 weeks	D3 capsules	Biotech Pharmacal Inc., Arizona, USA	HPLC-MRM (Becton Dickinson, Oxford, UK)	Bench Press (kg), Squat (kg), Vertical Jump (cm)
(35)	93.0 ± 19.0	95.0 ± 17.0	25.000	12 weeks	D3 tablets	Cal.D.Forte, PMS Healthcare, Auckland, NZ	LCMS-MS	Bench Press (kg), Bench Pull (kg), Weighted Reverse-Grip Chin-Up (kg)
(36)	48.5 ± 8.6	47.5 ± 16.2	35.000	8 weeks	D3 droplets	Vigantol, Merck, Germany	NR	SJ (cm), CMJ (cm)
(42)	27.3 ± 1.18	30.9 ± 1.95	35.000	4 weeks	D3 capsules	BioTech Pharmacal, Inc., Fayetteville, AR.	CLIA analyzer (DiaSorin Liaison, Italy)	CMJ
(37)	91.5 ± 4.3	101.8 ± 5.3	26.600	6 weeks	D2 powder	NR	HPLC-MS/MS	Vertical Jump (inches)
(39)	117.8 ± 9.2	126.5 ± 31.3	35.000	12 weeks	D3 gel capsules	Nature Made, Pharmavite LLC, Opelika, AL	CLIA analyzer (DiaSorin Liaison, Italy)	Bench Press (kg), Squat (kg), Deadlift (kg), Standing Broad Jump (cm), Vertical Jump (cm)
(50)	62.8 ± 12.5	69.5 ± 12.5	4.200	6 weeks	D2 capsules	NR	CLIA analyzer (Liaison, DiaSorin, USA)	Vertical Jump (watts)
(40)	47.4 ± 13.3	49.2 ± 25.4	21.000	12 weeks	D3 oral spray solution	BetterYou Ltd., Barnsley, UK	LCMS-MS	Vertical Jump (cm)
(41)	58 ± 23.4	59.0 ± 26.1	7.500	16 weeks	D3 tablets	NR	NR	CMJ (cm), Single Leg Hop (cm), Depth Jump (cm)

Data are mean ± standard deviation unless stated otherwise.

### Risk of bias in studies

**FIGURE 2:**
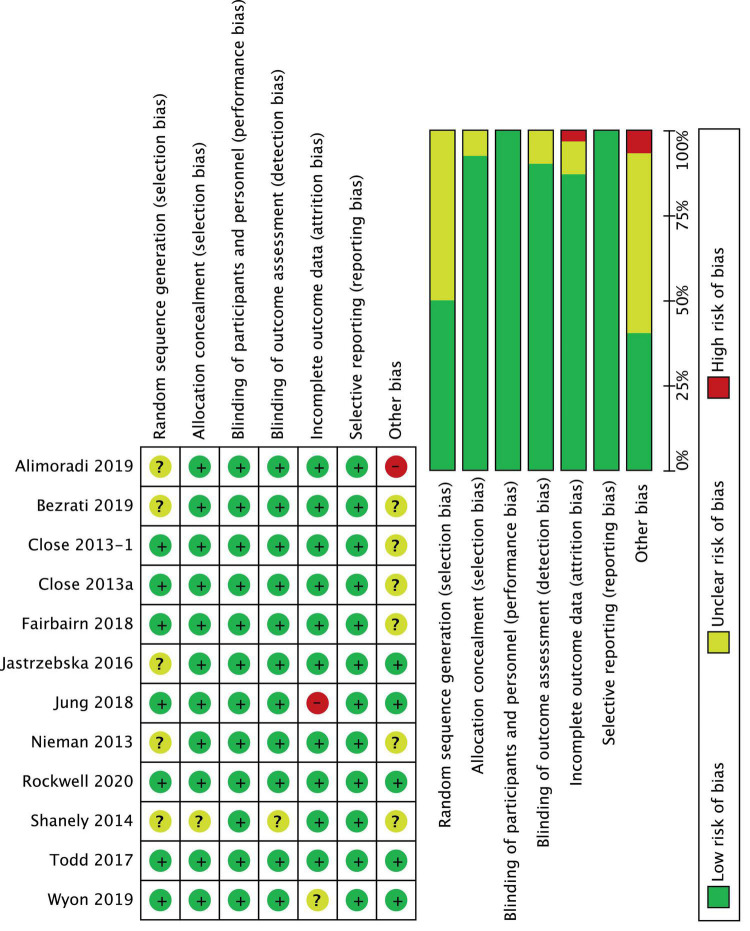
Risk of bias: review judgments about each risk of bias item for each included study.

**FIGURE 3:**
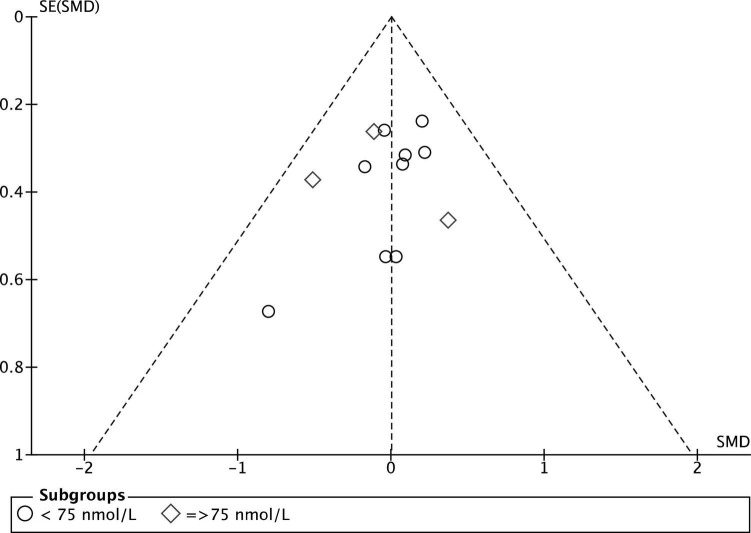
Funnel plot of the included RCTs for between groups mean difference of serum 25(OH)D concentration at baseline.

The quality evaluation of the included RCTs is presented in Figure 2. Overall, two studies presented a low risk of bias between all parameters (39, 40), eight studies presented “some concerns” (24, 33–36, 41, 50), and two trials were considered at “high risk” of bias (32, 42). Sequence generation was rated as “unclear risk” in 5 studies (32, 33, 36, 37, 50), because it was not described how the random sequence was generated. Moreover, in one trial (32) also the sample size calculation was not correctly specified, and data reported for vertical jump seemed ambiguous, which placed it at “high risk” of bias. Unclear risks were detected in the incomplete outcome domain in Wyon et al. (41) because there was no correspondence between what was reported in participants description and what was reported in summary Tables 1, 2. For the same domain, Jung et al.’s (42) study was considered at high risk of bias because it reported a loss of 20% of participants at follow-up. Additionally, in four studies (24, 35, 50) the sample population consisted of only male athletes or of only kids (33), which put them at “unclear risks” for other sources of bias. Finally, the funnel plot of the included RCTs for between-groups mean difference of serum 25(OH)D concentration at baseline is shown in Figure 3. The horizontal axis presents effect size, and the vertical axis presents standard error. The funnel plot showed the symmetry of studies, indicating low publication bias.

### Certainty of evidence

There were four outcome indicators in eleven studies. One was graded as moderate quality, two as low-quality and one as very-low-quality. The evidence was mostly downgraded owing to risk of bias, inconsistency and imprecision (Supplementary material 2).

### Results of serum 25(OH)D concentration

**FIGURE 4:**
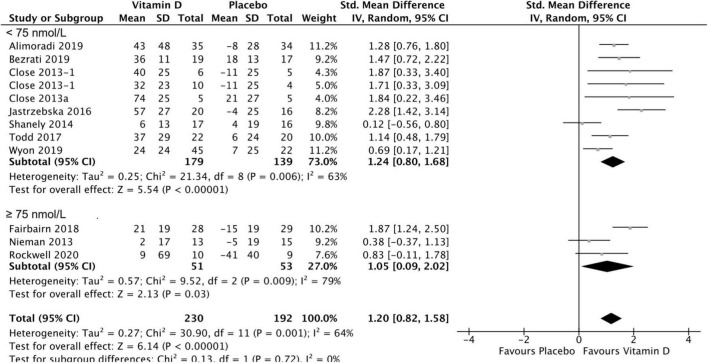
Forest plot for change from baseline in 25(OH)D serum concentration (nmol/L) for vitamin D and placebo groups. Nieman et al. (37) and Shanely (50) supplemented athletes with Vitamin D2, while all the remaining studies used Vitamin D3. Close et al. (24) reported multiple intervention groups: 20.000 and 40.000 IU vitamin D per week, so these have been separated out and reported as Close et al. (24, 34).

Twelve studies were included in the analysis of the effect of vitamin D supplementation on serum 25(OH)D concentration, with substantial heterogeneity detected among them (*I*^2^ = 70%, *p* = 0.0001). After conducting a sensitivity analysis by removing studies with higher/lower weights, Jung et al.’s (42) study was excluded from this meta-analysis, and no heterogeneity was detected among the remaining studies (*I*^2^ = 0%, *p* = 0.89). In fact, Jung’s medium study size and much smaller inner-study SDs skewed the effect size, thus making it different from other studies. There was no statistically significant difference in serum 25(OH)D concentration between treatment and placebo groups at baseline [SMD 0.00, 95% CI: (−0.19, 0.19); *p* = 1.00] overall, as well as stratified by baseline serum 25(OH)D level: SMD 0.05, 95% CI: (−0.18, 0.27), *p* = 0.69 in < 75 nmol/L subgroup, and SMD −0.13, 95% CI: (−0.54, 0.28), *p* = 0.53 in ≥ 75 nmol/L subgroup. When comparing the change from baseline in serum 25(OH)D concentration for vitamin D and placebo groups, the difference was statistically significant [SMD 1.24, 95% CI: (0.80, 1.68); *p* = 0.00001] in the subgroup of < 75 nmol/L, in the subgroup of ≥ 75 nmol/L, [SMD 1.05, 95% CI: (0.09, 2.02); *p* = 0.03] and overall [SMD 1.20, 95% CI: (0.82, 1.58); *p* = 0.0001] (Figure 4). Moreover, Table 3 shows the mean and SD of serum 25(OH)D concentration at baseline and follow-up, and the SMD within vitamin D and placebo groups. Stratifying by baseline serum 25(OH)D concentration, the pooled effect size of treatment groups increased [SMD 1.60, 95% CI: (1.05, 2.14), *p* < 0.0001] in < 75 nmol/L subgroup, and was estimated to be about one third the magnitude of increase in ≥ 75 nmol/L subgroup [SMD 0.50, 95% CI: (−0.19, 1.20), *p* = 0.16]. However, since the CIs (1.05, 2.14) vs. (−0.19, 1.2) overlapped, the difference between two stratified subgroups were not significant. In the placebo groups, a decrease of serum 25(OH)D concentration [SMD −0.68, 95% CI: (−1.09, −0.26)] was observed in ≥ 75 nmol/L subgroup and this reached statistical significance (*p* = 0.001).

**TABLE 3:** Baseline and follow-up serum 25(OH)D concentrations (nmol/L) for vitamin D and placebo groups.

References	Vitamin D					Placebo				
	**Follow-up**		**Baseline**			**Follow-up**		**Baseline**		
**< 75 nmol/L**	**Mean ± SD**	**N**	**Mean ± SD**	**N**	**IV, Random, 95% CI**	**Mean ± SD**	**N**	**Mean ± SD**	**N**	**IV, Random, 95% CI**
(32)	112.3 ± 49.5	35	68.8 ± 44.8	35	0.91 (0.42, 1.41)	53.3 ± 22.3	34	61.0 ± 31.8	35	−0.28 (−0.75, 0.20)
(33)	67.0 ± 10.7	19	31.0 ± 9.7	20	3.40 (2.39, 4.42)	48.3 ± 13.8	17	30.3 ± 11.3	20	1.41 (0.68, 2.14)
(24)	85.0 ± 10.0	10	53.0 ± 26.0	10	1.56 (0.53, 2.58)	41.0 ± 22.0	4	52.0 ± 27.0	5	−0.39 (−1.73, 0.95)
(34)	91.0 ± 24.0	6	51.0 ± 26.0	10	1.49 (0.32, 2.67)	41.0 ± 22.0	5	52.0 ± 27.0	5	−0.40 (−1.66, 0.86)
(34)	103.3 ± 25.0	5	29.0 ± 25.0	5	2.67 (0.72, 4.62)	74.0 ± 24.0	5	53.0 ± 29.0	5	0.71 (−0.59, 2.02)
(36)	106.3 ± 30.3	20	48.5 ± 8.6	20	2.54 1.69, 3.40)	43.5 ± 28.9	16	47.5 ± 16.2	16	−0.17 (−0.86, 0.53)
(50)	69.5 ± 12.5	17	62.8 ± 12.5	17	0.52 (0.16, 1.21)	62.0.17.5	16	65.5 ± 20.0	17	−0.18 (−0.87, 0.50)
(40)	83.7 ± 33.0	22	47.4 ± 13.3	22	1.42 (0.75, 2.09)	49.2 ± 25.4	20	43.1 ± 22.0	20	0.25 (−0.37, 0.87)
(41)	82.4 ± 23.8	45	58.0 ± 23.4	45	1.02 (0.58, 1.46)	66.3 ± 23.8	22	59.0 ± 26.1	22	0.29 (−0.31, 0.88)
**Subtotal (95% CI)**		**179**		**184**	**1.60 (1.05, 2.14)**		**139**		**145**	**0.15 (**−**0.24, 0.54)**
**≥ 75 nmol/L**										
(35)	114.0 ± 19.0	28	93.0 ± 19.0	29	1.09 (0.53, 1.65)	80.0 ± 21.0	29	95.0 ± 17.0	29	−0.77 (−1.31, −0.24)
(37)	93.5 ± 17.3	13	91.5 ± 16.7	15	0.11 (−0.63, 0.86)	96.5 ± 17.4	15	101.8 ± 20.5	15	−0.27 (−0.99, −0.45)
(39)	126.5 ± 78.3	10	117.8 ± 22.9	10	0.14 (−0.73, 1.02)	68.5 ± 45.8	9	110.0 ± 17.4	9	−1.14 (−2.16, −0.13)
**Subtotal (95% CI)**		**51**		**54**	**0.50 (**−**0.19, 1.20)**		**53**		**53**	−**0.68 (**−**1.09, 0.26)**
**Total (95% CI)**		**230**		**238**	**1.29 (0.83, 1.75)**		**192**		**198**	−**0.07 (**−**0.44, 0.30)**

### Results of 1 RM upper body test

**FIGURE 5:**
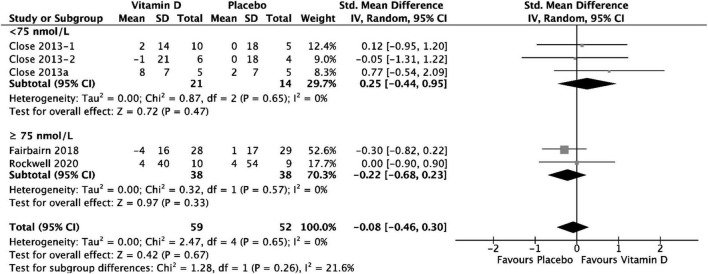
Forest plot for change from baseline in upper Body 1 RM tests (kg) for vitamin D and placebo groups. Upper body muscle strength was assessed by1 RM Bench Press test in all the included studies. Close et al. (24) reported multiple intervention groups: 20.000 and 40.000 IU vitamin D per week, so these have been separated out and reported as Close et al. (24, 34).

Group comparison consisting of four studies was used to assess the effect of vitamin D supplementation on upper body maximal strength, and substantial heterogeneity was detected (*I*^2^ = 61%, *p* = 0,04) at baseline. After removing Close et al. (34) during sensitivity analysis, *I*^2^ became 0%; however, since the overall effect size did not change [SMD 0.23, 95% CI: (−0.16, 0.62); *p* = 0.25], and the size of the study and the weight allocated to it were small (4.2%), the study by Close et al. (34) was still included in the analysis. A possible reason this study increased heterogeneity might be because its small size and much smaller inner-study SDs skewed the effect size. The analysis of all the included studies showed that there was no statistically significant difference in upper body 1 RM test values between treatment and placebo groups at baseline [SMD 0.11, 95% CI: (−0.27, 0.50); *p* = 0.56] overall, as well as stratified by baseline serum 25(OH)D level. When comparing the change in upper body 1 RM tests values from baseline between vitamin D and placebo groups, the results did not show any statistically significant differences [SMD 0.25, 95% CI: (−0.44, 0.95); *p* = 0.47] in the subgroup of < 75 nmol/L, in the subgroup of ≥ 75 nmol/L, [SMD −0.22, 95% CI: (−0.68, 0.23); *p* = 0.33] and overall (*p* = 0.67) (Figure 5). Among the included studies, Close et al. (34) reported a mean increase of 8 ± 7 kg and 2 ± 7 kg in 1 RM bench press for vitamin D and placebo groups, respectively. After a 12-week supplementation period with 20.000 IU\week or 40.000 IU/week vitamin D_3_, Close et al. (24) reported in another study, a mean increase of 2 ± 14 kg on 1 RM bench press, for the 20.000 IU/week group, and a mean decrease of 1 ± 21 kg on 1 RM bench press for the 40.000 IU/week group. Placebo group showed no increase for bench press. All data relative to the changes from baseline in 1RM tests are presented in Supplementary material 3.

### Results of 1 RM lower body test

**FIGURE 6:**
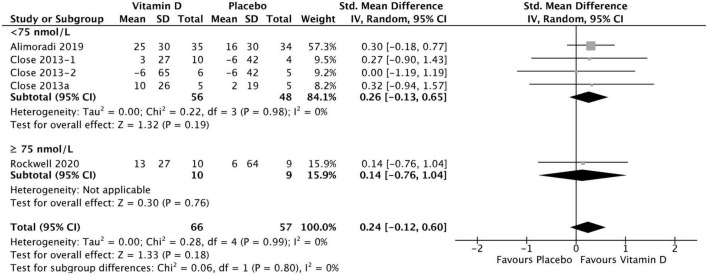
Forest plot for change from baseline in lower body 1 RM tests (kg) for vitamin D and placebo groups. Lower body muscle strength assessed by 1 RM Squat test in Close et al. (34) and Rockwell et al. (39), by 1 RM Leg Press test in Close et al. (24) and Alimoradi et al. (32). Close et al. (24) reported multiple intervention groups: 20.000 and 40.000 IU vitamin D per week, so these have been separated out and reported as Close et al. (24, 34).

Four studies were included in the analysis of the effect of vitamin D supplementation on lower body maximal strength, with no heterogeneity detected among them (*I*^2^ = 0%, *p* = 0.91). There was no statistically significant difference in lower body 1 RM test values between treatment and placebo groups at baseline [SMD −0.09, (95% CI: −0.44, 0.26), *p* = 0.62] overall, as well as stratified by baseline serum 25(OH)D level. When comparing the change from baseline in lower body 1 RM tests values for vitamin D and placebo groups, the difference was not statistically significant [SMD 0.26, 95% CI: (−0.13, 0.65), *p* = 0.19] in the < 75 nmol/L subgroup, as well as in the ≥ 75 nmol/L subgroup [SMD 0.14, 95% CI: (−0.76, 1.04), *p* = 0.76] and overall (*p* = 0.18) (Figure 6). Among the included studies, Alimoradi (32) reported a mean increase in 1 RM leg press test of 25 ± 30 kg in the treatment group and of 16 ± 30 kg in the placebo group, and this was the only study to show a statistically significant increase in both treatment and control groups. Close et al. (34) reported a mean increase of 10 ± 26 kg and 2 ± 19 kg in 1 RM squat for vitamin D and placebo groups, respectively. After a 12-week supplementation period with 20.000 IU\week or 40.000 IU/week vitamin D_3_, Close et al. (24) reported in another study, a mean increase 3 ± 27 kg on 1 RM leg press for the 20.000 IU/week group, and a mean decrease of 6 ± 65 kg on1 RM leg press for the 40.000 IU/week group. Placebo group showed a mean decrease of 6 ± 42 kg for 1 RM leg press. All data relative to the changes from baseline in 1RM tests are presented in Supplementary material 3.

### Results of muscle power test

**FIGURE 7:**
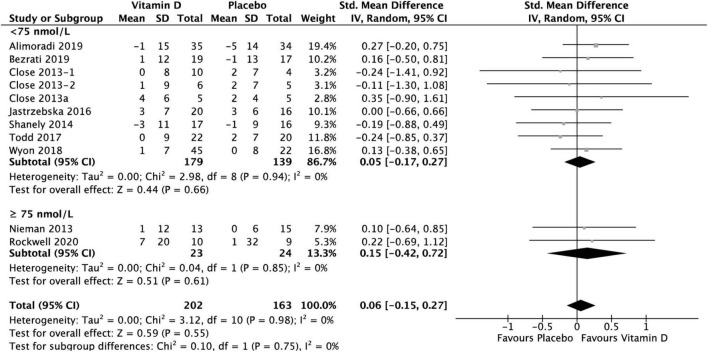
Forest plot for change from baseline in muscle power tests (cm) for vitamin D and placebo groups. Muscle power was assessed by CMJ test in Wyon et al. (41) and by Vertical Jump test in the remaining studies. Close et al. (24) reported multiple intervention groups: 20.000 and 40.000 IU vitamin D per week, so these have been separated out and reported as Close et al. (24, 34).

Group comparison consisting of ten studies was used to assess the effect of vitamin D supplementation on muscle power, with no heterogeneity detected among them (*I*^2^ = 0%, *p* = 0.46). The difference between vitamin D and placebo groups at baseline for muscle power test was not statistically significant over all: SMD −0.03, 95% CI: (−0.24, 0.18), *p* = 0.78. When comparing the change in muscle power tests from baseline between vitamin D and placebo groups, the results did not show any statistically significant differences overall [SMD 0.06, 95% CI: (−0.15, 0.27); *p* = 0.55], as well as in the subgroup of < 75 nmol/L [SMD 0.05, 95% CI: (−0.17, 0.27); *p* = 0.66], and in the subgroup of ≥ 75 nmol/L, [SMD 0.15, 95% CI: (−0.42, 0.72); *p* = 0.61] (Figure 7). However, data for vertical jump were reported in only two studies for the ≥ 75 nmol/L subgroup: Nieman et al. (37) reported a mean change of 1 ± 12 cm in treatment group and no change in placebo group, while Rockwell et al. (39) reported a mean change of 7 ± 20 cm and 1 ± 32 cm in treatment and placebo group, respectively. All data relative to the changes from baseline in muscle power tests are presented in Supplementary material 3.

## Discussion

The aim of the present meta-analysis was to assess the effect of vitamin D supplementation on upper and lower body maximal strength and power in athletic populations. The findings demonstrate that supplementation significantly increased serum 25(OH)D concentration regardless of the baseline concentration, compared to placebo. When the baseline serum 25(OH)D concentration was < 75 nmol/L, the intervention was estimated to be three times more effective than the stratum with baseline serum 25(OH)D concentration ≥ 75 nmol/L (Table 3).

However, regardless of the baseline serum 25(OH)D concentration, there was no effect of treatment on muscle strength of upper and lower body. Finally, vitamin D supplementation had no statistically significant effect on muscle power regardless of the baseline serum 25(OH)D concentration. Nevertheless, according to the GRADE system, we found only one moderate quality outcome, two low-quality outcomes and one very-low-quality outcome. The highest downgrading factor was the risk of bias, mainly due to RCTs with unclear or missing randomization, blinding and allocation concealment. Therefore, researchers should pay more attention to the design and implementation processes in the future studies.

### Effect of supplementation on vitamin D status

The results of the included RCTs suggest that a supplementation with a minimum daily dosage ≥ 600 IU of vitamin D_2_ for 6 weeks (50) or ≥ 30.000 IU for 4 days (41), was helpful to increase total serum 25(OH)D when baseline concentration was < 75 nmol/L, in these cohorts of athletes during autumn\winter. However, among the included studies, a minimum dose of 20.000 IU/week of vitamin D_3_ for 12 weeks (24) was necessary to reach a serum 25(OH)D concentration ≥ 75 nmol/L. This result aligns with the findings of Farrokhyar et al. (8) that suggest a supplementation of > 3,000 IU/day in fall and winter is necessary for athletes to achieve sufficiency during wintertime, when the sun exposure is minimal. When baseline concentration was ≥ 75 nmol/L, a supplementation with a daily dosage ≥ 3,800 IU of vitamin D_2_ for 6 weeks (37) or a weekly dosage of 25.000 IU of vitamin D_3_ (35) was helpful in maintaining vitamin D sufficiency. These results showed that a higher Vitamin D dosage was necessary to maintain vitamin D sufficiency compared to the findings of Holick et al. (51) which suggested a maintenance therapy of 1,500–2,000 IU/d of Vitamin D_2_ or D_3_, after a sufficient serum 25(OH)D concentration has been reached. Interestingly, a statistically significant lowering (*p* = 0.001) of serum 25(OH)D concentration at follow-up in the ≥ 75 nmol/L subgroup of the placebo group was observed (Table 3). The season of the year, larger body (or fat) mass, insufficient dietary intake, poor sunlight exposure, dark skin color and participation in indoor activities might have been possible reasons for this decline in serum 25(OH)D concentration in athletes (52). When a single bolus protocol was used, a dose of 200.00 IU of vitamin D was not helpful in reaching vitamin D sufficiency (41). All the included RCTs assumed that vitamin D status can be affected only by vitamin D_3_ or D_2_ oral supplementation; however, there is now evidence that also skeletal muscle cells might play a role in this regard, by storing vitamin D and using it in winter when sunlight exposure is minimal (53). Future studies therefore, should attempt to measure also the vitamin D stored in the muscle cells to gain a complete insight into interactions on vitamin D on muscle.

### Effect of vitamin D supplementation on muscle strength

In contrast to the observation reported by Zhang et al. (16) in a previous meta-analysis, where they found that vitamin D supplementation had an effect on increasing only lower limbs muscle strength in athletes, the results of the current meta-analysis showed no statistically significant effect on both upper and lower body muscle strength. This might be associated with several factors. First, the RCTs included in the present report used only 1 RM tests in multi-joints exercises to assess maximal strength, providing more consistent protocols across the studies than the ones used in the previous reviews. Second, the hypothesis that muscle strength gains might be different when comparing upper and lower body has no general consensus. The studies that tried to address this upper to lower body question varied in the strength training protocols applied, specifically in the number of exercises and sets performed for different muscles, the types of tests used to assess muscle strength, and the type of population recruited (54). Therefore, the hypothesis around potential upper to lower body differences in response to vitamin D supplementation, must be further investigated in athletic populations.

Even if a significant increase in serum 25(OH)D concentration was reported in all the included studies, the magnitude of changes in most 1RM test values did not entirely correspond to the increase in concentration in the < 75 nmol/L subgroup. Small changes can be expected in highly trained athletes that typically have minimal margins of improvements. However, even if the population in the included studies was mostly represented by professional and semi-professional team-sport athletes, observing the baseline values of the various 1 RM tests, it could be hypothesized that they were not advanced strength-trained athletes. A larger increase in terms of strength could have been expected after a well-periodized training program. In fact, a well-designed resistance training program is supposed to lead to an increase in muscle strength as a result of early neuromuscular adaptations, followed by increases in muscle cross sectional area (CSA), and alteration in connective tissue stiffness (55). The early neuromuscular adaptations occurring after strength training are related to the increased voluntary activation of muscles, increased motor unit synchronization, decreased in co-activation of antagonist muscles, and increased rate of torque development (56, 57). At a muscle fiber level, the main adaptations observed after strength training, besides an increase in muscle cross-sectional area, are fiber type conversions and an increase in muscle fiber peak power (58, 59). The adaptations to resistance training are generally evident after 8 to 12 weeks, even if some studies have observed increases after only 2 to 4 weeks, probably as consequence of early neural adaptation (55). Larger increase in muscle strength therefore, could have been expected in the included studies, even in the case of team-sport athletes that combine strength and aerobic training in the same program. This combination, known as concurrent training, does not interfere with strength gains, as long as the aerobic training is sport-specific and therefore performed in the form of high intensity interval training (HIIT) (60). Considering that none of the studies showed the content of the strength training programs used by the athletes during the intervention period, it is difficult to establish if the small changes in 1 RM test values were due to a lack of effect of vitamin D on muscle strength when baseline serum 25(OH)D concentration is < 75 nmol/L, to the lack of a training effect induced by the programme, or both. Furthermore, a large individual variance was evident in the confidence interval for strength gains, suggesting that some athletes have a large response to supplementation, training or a combination of both, while others having no response. This variance, that is very common among team sport athletes when it comes to assessing non-specific sport skills, is interesting and should be investigated in future well-controlled RCTs that document the training programmes implemented. Finally, the small decrease reported in upper body muscle strength in the ≥ 75 nmol/L subgroup might also indicate that vitamin D supplementation is not helpful in increasing maximal strength when serum 25(OH)D concentration is ≥ 75 nmol/L.

### Effect of vitamin D supplementation on muscle power

No significant improvement was observed in muscle power in either baseline serum 25(OH)D concentration subgroup. However, possible limitations of this observation are that the result might have been confounded by the significant lowering of serum 25(OH)D concentration at follow-up in the placebo group (*p* = 0.001) (Table 3), and that data for vertical jump were reported in only two studies for the ≥ 75 nmol/L subgroup (37, 39). As was observed for upper and lower body 1 RM tests, no significant changes have been reported also in these studies. However, since none of the included studies reported the training program, it is difficult to tell if reaching a serum concentration ≥ 75 nmol/l has no effect on muscle power or if an increase could have been expected after a well-planned training program. In fact, when periodization principles are correctly applied, strength training results in faster power improvement and allows athletes to reach higher power levels (61).

### Strengths and limitations

This study is the first meta-analysis to quantitatively assess the effects of vitamin D supplementation on muscle strength in athletes only by 1 RM tests, considered the gold standard for measuring maximum strength in non-laboratory environments (18), and a valid and reliable method to assess muscle strength changes regardless of muscle group location or gender (62). Moreover, data stratification by serum 25(OH)D concentration < 75 nmol/L and ≥ 75 nmol/L was deemed necessary if trying to detect small effects on muscle strength and power, but this did not reveal any significant effects.

The present study presents also some limitations. First, there was a large variation in the populations analyzed in the included RCTs, with different sport activities, competition levels, nationalities, and training latitudes, and in the supplementation protocols, including dosage, frequency, duration and timing of the intervention. Even athletes race or ethnicity was not mentioned in most of the included RCTs. Black athletes often present with deficient 25(OH)D concentration compared to white athletes. These differences in 25(OH)D levels are likely related to polymorphism in VDBP, resulting in lower concentrations of VDBP and total 25(OH)D, but higher concentration of free vitamin D in black athletes (10). Using 25(OH)D as a marker of vitamin D status therefore, might represent a major limitation. Moreover, we used the Cochrane Risk of Bias Tool 1 to assess the quality of the included studies. We recognize that the more recent version, Risk of Bias Tool 2, might be more stringent and that studies might end up at being at higher risk of bias if analyzed differently. Second, potentially relevant studies may have been missed due to the limitation of the search strategy only to English language. Third, the sample population in most studies consisted of only male athletes who have higher 25(OH)D concentration than female athletes due to adiposity and BMI being inversely related to serum 25(OH)D concentration (34). All these factors were most likely the cause of between-study heterogeneity in some strata. Fourth, despite a total of 436 subjects in 11 studies represented a larger overall sample size larger than previous meta-analysis, some of the sub-analysis presented low outcome numbers. Finally, the standard deviations of the changes from baseline in 1 RM and power tests values were missing, and they were calculated (23). For this analysis it was assumed that the correlation between baseline and follow-up measurements was *p* = 0.5 (63), and this could be a source of error.

## Conclusion

The present meta-analysis demonstrated that there is not strong evidence for an effect of vitamin D supplementation on improving maximum strength and power in athletic populations. However, given the restricted number of studies included in this meta-analysis, the large individual variance evident in the coefficient intervals for strength and power gains, and the limitation of using 25(OH)D as a marker of vitamin D status, further RCTs are needed to investigate the effect of Vitamin D supplementation in athletes. Highly trained athletes typically have minimal margins for improvement compared to the general population; thus, future studies should involve athletic population with larger sample size, use 1,25(OH)D concertation to assess vitamin D status, control for gender differences to determine if there is an optimal vitamin D concentration for female athletes, and report in details the training program the athletes did before and during the supplementation period. Moreover, since the initial increases in force production with resistance training are fast and thought to be primarily underpinned by neural adaptations (64), studies duration should be long enough (8–12 weeks) to reduce the possibility that any strength improvement might be consequence of only early neural adaptations rather than the effect of the intervention.

### Practical implications

While athletes with a vitamin D status < 75 nmol/L may consider vitamin D supplementation to correct vitamin D deficiency, reaching a serum 25(OH)D concentration ≥ 75 nmol/L does not enhance maximal strength and power.

## Data availability statement

The original contributions presented in this study are included in the article/Supplementary material, further inquiries can be directed to the corresponding author.

## Author contributions

All authors listed have made a substantial, direct, and intellectual contribution to the work, and approved it for publication.
