# Supplementary material for: Effects of vitamin D supplementation on maximal strength and power in athletes: a systematic review and meta-analysis of randomized controlled trials
Source: Front Nutr. 2023 Sep 29;10:1163313. doi: 10.3389/fnut.2023.1163313 (PMC10570740; doi:10.3389/fnut.2023.1163313)
Supplement: Supplementary file 1 [file Data_Sheet_1.PDF]

Identification

	Query Box	Filters	Results	TOTAL
**SPORT Discus**	Vitamin D OR Ergocalciferol OR Cholecalciferol AND military personnel OR Military Training OR Tactical Training	-	17	**250**
	Vitamin D OR Ergocalciferol OR Cholecalciferol AND Maximal Strength OR Muscle Power	-	9	
	Vitamin D OR Ergocalciferol OR Cholecalciferol AND Bench Press OR Squat OR Leg Press OR Deadlift OR Jump	-	38	
	Vitamin D OR Ergocalciferol OR Cholecalciferol AND Swimming OR Soccer OR Rugby OR Basketball OR Rowing OR Running OR Football OR Skiing OR Tennis OR Cyclist OR Team Sports	-	186	
**PubMed**	(Vitamin D OR Cholecalciferol OR Ergocalciferol) AND (military personnel OR Military Training OR Tactical Training)	Humans	133	**592**
	((Vitamin D) OR (Cholecalciferol) OR (Ergocalciferol)) AND ((Maximal Strength) OR (Muscle Power))	Humans	177	
	((Vitamin D) OR (Cholecalciferol) OR (Ergocalciferol)) AND ((Bench Press) OR (Squat) OR (Leg Press) OR (Deadlift) OR (Jump))	Humans	83	
	((Vitamin D) OR (Cholecalciferol) OR (Ergocalciferol)) AND ((Swimming) OR (Soccer) OR (Rugby) OR (Basketball) OR (Rowing) OR (Running) OR (Football) OR (Skiing) OR (Tennis) OR (Cyclist) OR (Team Sports))	Humans	199	
**Cochrane Library**	Vitamin D OR Cholecalciferol OR Ergocalciferol in Title Abstract Keyword AND Military Personnel OR Military Training OR Tactical Training in Title Abstract Keyword	Trials	46	**663**
	Vitamin D OR Cholecalciferol OR Ergocalciferol in Title Abstract Keyword AND Maximal Strength OR Muscle Power in Title Abstract Keyword	Trials	191	
	Vitamin D OR Cholecalciferol OR Ergocalciferol in Title Abstract Keyword AND Bench Press OR Leg Press OR Squat OR Deadlift OR Jump in Title Abstract Keyword	Triials	110	
	Vitamin D OR Cholecalciferol OR Ergocalciferol in Title Abstract Keyword AND Swimming OR Soccer OR Rugby OR Basketball OR Rowing OR Running OR Football OR Skiing OR Tennis OR Cyclist OR Team Sports in Title Abstract Keyword	Trials	316	
**WoS**	**TITLE:** (Vitamin D OR Cholecalciferol OR Ergocalciferol) *AND* **TOPIC:** (Military Personnell OR Military Training OR Tactical Training)	All Databases	24	**589**
	**TITLE:** (Vitamin D OR Cholecalciferol OR Ergocalciferol) *AND* **TOPIC:** (Leg Press OR Squat OR Bench Press OR Deadlift OR Jump)	All Databases	64	
	**TITLE:** (Vitamin D OR Cholecalciferol OR Ergocalciferol) *AND* **TOPIC:** (Maximal Strength OR Muscle Power)	All Databases	138	
	**TITLE:** (Vitamin D OR Cholecalciferol OR Ergocalciferol) *AND* **TOPIC:** (Swimming OR Soccer OR Rugby OR Basketball OR Rowing OR Running OR Fotball OR Skiing OR Tennis OR Cyclcist OR Team Sports)	All Databases	363	
**Manual Cross referencing**	Wyon et al., 2016		1	4
	Ercan 2019		1	
	Shanely et al., 2014		1	
	Mitchel, 2013		1	

TOTAL	Duplicates Removed	All references
**2098**	**592**	**1506**

Question: Vitamin D supplementation compared to Placebo for increasing maximal strength and power in athletes
Setting: Professional athletes

Certainty assessment							№ of patients		Effect		Certainty	Importance
№ of studies	Study design	Risk of bias	Inconsistency	Indirectness	Imprecision	Other considerations	Vitamin D supplementation	Placebo	Relative (95% CI)	Absolute (95% CI)		
**Serum 25(OH)D**												
11	randomised trials	serious[1,2,3,4,5,6,a,b,c,d,e,f]	not serious	not serious	serious[2,6,7,8,9,a,g]	none	230	192	-	SMD **1.19 higher** (0.81 higher to 1.57 higher)	⊕⊕◯◯ Low	
**1 RM Upper Body**												
4	randomised trials	serious[3,5,c,d]	not serious	not serious	not serious	none	59	52	-	SMD **0.08 lower** (0.46 lower to 0.3 higher)	⊕⊕⊕◯ Moderate	
**1 RM Lower Body**												
4	randomised trials	serious[3,5,6,a,c,d]	not serious	not serious	serious[6,f]	none	66	57	-	SMD **0.24 higher** (0.12 lower to 0.6 higher)	⊕⊕◯◯ Low	
**Muscle Power**												
10	randomised trials	serious[2,3,4,5,6,7,8,9,a,b,c,d,e,g,h,i]	serious	not serious	serious[6,f]	none	202	163	-	SMD **0.06 higher** (0.15 lower to 0.27 higher)	⊕◯◯◯ Very low	

CI: confidence interval; **SMD:** standardised mean difference

Explanations

a. It's not clear how the random sequence was generated.
b. Population consisted of only children.
c. Very small small sample size
d. Population consisted of only male athletes.
e. It is not clear if the allocation was concealed
f. Sample size calculation has not been clearly specified
g. There is no correspondence between what is reported in participants description and what is reported in the tables
h. Data reported for Vertical Jump need to be clarified
i. It is not specified who assessed perfomance and if they were blinded

References

1.Fairbairn, , K., A.;,Ceelen, I., J.,M.;,Skeaff, C., M.;,Cameron, C., M.;,Perry, T., L.. Vitamin D(3) Supplementation Does Not Improve Sprint Performance in Professional Rugby Players: A Randomized, Placebo-Controlled, Double-Blind Intervention Study. Int J Sport Nutr Exerc Metab; 2018.
2.Shanely, , R., A.;,Nieman, D., C.;,Knab, A., M.;,Gillitt, N., D.;,Meaney, M., P.;,Jin, F.;, Sha, W.;, Cialdella-Kam, L., . Influence of vitamin D mushroom powder supplementation on exercise-induced muscle damage in vitamin D insufficient high school athletes. J Sports Sci; 2014.
3.Close, , G., L.;,Russell, J.;, Cobley, J., N.;,Owens, D., J.;,Wilson, G.;, Gregson, W.;, Fraser, W., D.;,Morton, J., P.. Assessment of vitamin D concentration in non-supplemented professional athletes and healthy adults during the winter months in the UK: implications for skeletal muscle function. Journal of Sports Sciences; 2013.
4.Bezrati, , I.;, Ben,Fradj, M., K.;,Hammami, R.;, Ouerghi, N.;, Padulo, J.;, Feki, M., . A single mega dose of vitamin D3 improves selected physical variables in vitamin D insufficient young amateur soccer players: a randomized controlled trial. Physiologie appliquee, nutrition et metabolisme [Applied physiology, nutrition, and metabolism]; 2019.
5.Close, , Graeme, L.;,Leckey, Jill;, Patterson, Marcelle;, Bradley, Warren;, Owens, Daniel, J.;,Fraser, William, D.;,Morton, James, P.. The effects of vitamin D3 supplementation on serum total 25[OH]D concentration and physical performance: a randomised dose--response study. British Journal of Sports Medicine; 2013.
6.Alimoradi, , Karamollah;, Nikooyeh, Bahareh;, Ravasi, Ali, Asghar;,Zahedirad, Maliheh;, Shariatzadeh, Nastaran;, Kalayi, Ali;, Neyestani, Tirang, Reza. Efficacy of Vitamin D Supplementation in Physical Performance of Iranian Elite Athletes. International Journal of Preventive Medicine; 2019.
7.Wyon, , Matthew, A.;,Wolman, Roger;, Kolokythas, Nicolas;, Sheriff, Karen;, Galloway, Shaun;, Mattiussi, Adam, . Effect of Vitamin D on Muscle Function and Injury in Elite Adolescent Dancers: A Randomized Double-Blind Study. International Journal of Sports Physiology and Performance; 2019.
8.Nieman, , D., C.;,Gillitt, N., D.;,Shanely, R., A.;,Dew, D.;, Meaney, M., P.;,Luo, B., . Vitamin D2 supplementation amplifies eccentric exercise-induced muscle damage in NASCAR pit crew athletes. Nutrients; 2013.
9.Jastrzebska, , Maria;, Kaczmarczyk, Mariusz;, Jastrzebski, Zbigniew, . Effect of Vitamin D Supplementation on Training Adaptation in Well-Trained Soccer Players. Journal of Strength and Conditioning Research; 2016.

1 RM UPPER VIT D							MEAN CHANGE	SD CHANGE	Corr Coeff
	Follow up			Baseline					
< 75 nmol/L	Mean	SD	N	Mean	SD	N			
Close 2013	92	15	10	90	13	10	2	14	0,5
Close 2013	90	20	6	91	22	10	-1	21	
Close 2013a	94	8	5	86	4	5	8	7	
≥ 75 nmol/L									
Fairbairn 2018	122	15	28	126	17	29	-4	16	
Rockwell 2020	68	46	10	64	28	10	4	40	

1 RM UPPER PLA							MEAN CHANGE	SD CHANGE
	Follow up			Baseline				
< 75 nmol/L	Mean	SD	N	Mean	SD	N		
Close 2013	79	18	5	79	17	5	0	18
Close 2013	79	18	4	79	17	5	0	18
Close 2013a	99	8	5	97	4	5	2	7
≥ 75 nmol/L								
Fairbairn 2018	123	16	29	122	17	29	1	17
Rockwell 2020	80	61	9	76	41	9	4	54

1 RM LOWER VIT D							MEAN CHANGE	SD CHANGE
	Follow up			Baseline				
< 75 nmol/L	Mean	SD	N	Mean	SD	N		
Alimoradi 2019	150	32	35	125	27	35	25	30
Close 2013	198	28	10	195	26	10	3	27
Close 2013	198	63	6	204	66	10	-6	65
Close 2013a	147	28	5	137	23	5	10	26
≥ 75 nmol/L								
Rockwell 2020	94	23	10	81	29	10	13	27

1 RM LOWER PLA							MEAN CHANGE	SD CHANGE
	Follow up			Baseline				
< 75 nmol/L	Mean	SD	N	Mean	SD	N		
Alimoradi 2019	145	32	34	129	28	35	16	30
Close 2013	181	43	5	187	41	5	-6	42
Close 2013	181	43	4	187	41	4	-6	42
Close 2013a	145	18	5	143	19	5	2	19
≥ 75 nmol/L								
Rockwell 2020	100	54	9	94	71	9	6	64

POWER VIT D							MEAN CHANGE	SD CHANGE
	Follow up			Baseline				
< 75 nmol/L	Mean	SD	N	Mean	SD	N		
Alimoradi 2019	78	13	35	79	17	35	-1	15
Bezrati 2019	12	10	19	11	14	20	1	12
Close 2013	48	10	6	47	7	10	1	9
Close 2013	49	9	10	49	7	10	0	8
Close 2013a	56	5	5	52	7	5	4	6
Jastrzebska 2016	39	8	20	36	5	20	3	7
Jung 2018	57	5	20	54	5	22	3	5
Shanely 2014	58	11	17	61	10	17	-3	11
Todd 2017	32	9	22	32	8	22	0	9
Wyon 2019	32	7	45	31	7	45	1	7
≥ 75 nmol/L								
Nieman 2013	77	12	13	76	12	15	1	12
Rockwell 2020	58	23	10	51	8	10	7	20

POWER PLA							MEAN CHANGE	SD CHANGE
	Follow Up			Baseline				
< 75 nmol/L	Mean	SD	N	Mean	SD	N		
Alimoradi 2019	80	13	34	85	15	35	-5	14
Bezrati 2019	11	12	17	12	14	20	-1	13
Close 2013	48	6	5	46	7	5	2	7
Close 2013	48	6	4	46	7	5	2	7
Close 2013a	53	3	5	51	4	5	2	4
Jastrzebska 2016	39	7	16	35	5	16	4	6
Jung 2018	55	4	15	54	5	22	1	5
Shanely 2014	62	9	16	63	9	17	-1	9
Todd 2017	29	7	20	27	6	20	2	7
Wyon 2019	33	7	22	33	8	22	0	8
≥ 75 nmol/L								
Nieman 2013	74	5	15	74	6	15	0	6
Rockwell 2020	59	36	9	58	27	9	1	32

SERUM 25(OH)D VIT D							MEAN CHANGE	SD CHANGE
	Follow Up			Baseline				
< 75 nmol/L	Mean	SD	N	Mean	SD	N		
Alimoradi 2019	112	50	35	69	45	35	43	48
Bezrati 2019	67	11	19	31	10	20	36	11
Close 2013	91	24	6	51	26	10	40	25
Close 2013	85	10	10	53	26	10	32	23
Close 2013a	103	25	5	29	25	5	74	25
Jastrzebska 2016	106	30	20	49	9	20	57	27
Shanely 2014	69	13	17	63	13	17	6	13
Todd 2017	84	33	22	47	13	22	37	29
Wyon 2019	82	24	45	58	23	45	24	24
≥ 75 nmol/L								
Fairbairn 2018	114	19	28	93	19	29	21	19
Nieman 2013	94	17	13	92	17	15	2	17
Rockwell 2020	127	78	10	118	23	10	9	69

SERUM 25(OH)D PLA							MEAN CHANGE	SD CHANGE
	Follow Up			Baseline				
< 75 nmol/L	Mean	SD	N	Mean	SD	N		
Alimoradi 2019	53	22	34	61	32	35	-8	28
Bezrati 2019	48	13	17	30	11	20	18	12
Close 2013	41	22	5	52	27	5	-11	25
Close 2013	41	22	4	52	27	5	-11	25
Close 2013a	74	24	5	53	29	5	21	27
Jastrzebska 2016	44	29	16	48	16	16	-4	25
Shanely 2014	62	18	16	66	20	17	-4	19
Todd 2017	49	25	20	43	22	20	6	24
Wyon 2019	66	24	22	59	26	22	7	25
≥ 75 nmol/L								
Fairbairn 2018	80	21	29	95	17	29	-15	19
Nieman 2013	97	17	15	102	21	15	-5	19
Rockwell 2020	69	46	9	110	17	9	-41	40
	0,91	22		-0,28	32			
